# *In vitro* and *in vivo* Inhibitory Activity of NADPH Against the AmpC BER Class C β-Lactamase

**DOI:** 10.3389/fcimb.2018.00441

**Published:** 2018-12-21

**Authors:** Jung-Hyun Na, Tae Hee Lee, Soo-Bong Park, Min-Kyu Kim, Bo-Gyeong Jeong, Kyung Min Chung, Sun-Shin Cha

**Affiliations:** ^1^Department of Chemistry and Nanoscience, Ewha Womans University, Seoul, South Korea; ^2^Department of Microbiology and Immunology, Chonbuk National University Medical School, Jeonju, South Korea; ^3^Institute for Medical Science, Chonbuk National University Medical School, Jeonju, South Korea; ^4^Biotechnology Research Division, Korea Atomic Energy Research Institute, Jeongeup, South Korea; ^5^Department of Radiation Biotechnology and Applied Radioisotope Science, University of Science and Technology, Daejeon, South Korea

**Keywords:** antimicrobial resistance, class C β-lactamase, AmpC BER, NADPH, β-lactamase inhibitors, mouse infection model

## Abstract

β-Lactamase-mediated resistance to β-lactam antibiotics has been significantly threatening the efficacy of these clinically important antibacterial drugs. Although some β-lactamase inhibitors are prescribed in combination with β-lactam antibiotics to overcome this resistance, the emergence of enzymes resistant to current inhibitors necessitates the development of novel β-lactamase inhibitors. In this study, we evaluated the inhibitory effect of dinucleotides on an extended-spectrum class C β-lactamase, AmpC BER. Of the dinucleotides tested, NADPH, a cellular metabolite, decreased the nitrocefin-hydrolyzing activity of the enzyme with a *K*_*i*_ value of 103 μM in a non-covalent competitive manner. In addition, the dissociation constant (*K*_D_) between AmpC BER and NADPH was measured to be 40 μM. According to our *in vitro* susceptibility study based on growth curves, NADPH restored the antibacterial activity of ceftazidime against a ceftazidime-resistant *Escherichia coli* BER strain producing AmpC BER. Remarkably, a single dose of combinatory treatment with NADPH and ceftazidime conferred marked therapeutic efficacy (100% survival rate) in a mouse model infected by the *E. coli* BER strain although NADPH or ceftazidime alone failed to prevent the lethal bacterial infection. These results may offer the potential of the dinucleotide scaffold for the development of novel β-lactamase inhibitors.

## Introduction

The β-lactam antibiotics are the mainstay in the treatment of serious bacterial infections in clinical use (Liu et al., 2015). However, the prevalence of β-lactamases produced by pathogenic bacteria has been the major obstacle to cure bacterial infections because these enzymes hydrolyze the essential β-lactam ring for antibiotic efficacy. In addition, the recent overuse of antibiotics in human medicine and agriculture has promoted alterations and mutations within β-lactamases (Fair and Tor, 2014), which extends their substrate spectrum and reduces the susceptibility of bacteria to the most clinically important antibiotics, including penicillin derivatives, cephalosporins, monobactams, and carbapenems (Bush and Jacoby, 2010).

In the past decades, a variety of β-lactamases have been identified, classified into four classes (Ambler classes A, B, C, and D) based on their amino acid sequences (Hall and Barlow, 2005), and investigated to overcome drawback of their drug resistances. One approach to preserve the utility of β-lactam antibiotics is to develop β-lactamase inhibitors that maintain the efficacy of β-lactam antibiotics by reducing the activity of β-lactamases (Davies, 1994). Several β-lactamase inhibitors, including β-lactam inhibitors (clavulanate, sulbactam, and tazobactam) and non-β-lactam inhibitors (avibactam and vaborbactam), have been developed and clinically used to treat bacterial infections in combination with β-lactam antibiotics (e.g., amoxicillin/clavulanate, ticarcillin/clavulanate, ampicillin/sulbactam, cefoperazone/sulbactam, piperacillin/tazobactam, ceftazidime/avibactam, and meropenem/vaborbactam) (Harris et al., 2015; Cho et al., 2018). However, clavulanate, sulbactam, and tazobactam display inhibitory activity only against class A β-lactamases with much less or no effect on class B, C, and D β-lactamases although avibactam is active against class A, C, and some D β-lactamases and vaborbactam inhibits class A and C β-lactamases. (Bush, 1988; Buynak, 2006; Drawz and Bonomo, 2010; Ehmann et al., 2013; Cho et al., 2018). Furthermore, the ongoing emergence and dissemination of novel β-lactamases that are resistant to currently available β-lactamase inhibitors and β-lactam antibiotic/β-lactamase inhibitor combinations (Shields et al., 2017) highlights the need to develop new β-lactamase inhibitors.

Recently, it has been demonstrated that mononucleotides including adenosine 5′-(*P*-acetyl) monophosphate (acAMP), guanosine monophosphate (GMP), and inosine monophosphate (IMP) can exert inhibitory activities on class C β-lactamases (Kim et al., 2017; Na et al., 2017), which was inspired by the observation of the adenylylated nucleophilic serine in the active sites of Fox-4 and AmpC BER (Lefurgy et al., 2015; Kim et al., 2017). Furthermore, the combination of acAMP and ceftazidime, a third-generation cephalosporin, significantly reduced the growth of antibiotic resistant bacteria (Kim et al., 2017). These results suggest that nucleotide scaffolds could be useful candidates to develop novel β-lactamase inhibitors. In this study, we describe the inhibitory activity of the reduced form of nicotinamide adenine dinucleotide 2′-phosphate (NADPH) against class C β-lactamases. Through *in vitro* assays and a mouse infection model, we characterized the inhibitory mechanism and effect of NADPH on an extended-spectrum class C β-lactamase, AmpC BER. Overall, these studies might facilitate the identification and design of new and high effective inhibitors targeting class C β-lactamases.

## Materials and Methods

### Cloning, Expression, and Purification of Class C β-Lactamases

Cloning of class C β-lactamases (ACC-1, AmpC BER, AmpC EC2, CMY-2, and CMY-10) were performed as described previously (Kim et al., 2017). The constructs were transformed into the *E. coli* strain BL21 (DE3). Expression of recombinant proteins was induced by 1 mM isopropyl β-D-1-thiogalactopyranoside for 18 h at 20°C when the optical density at 600 nm of the transformed cells reached ~0.5. After induction, the cells were harvested by centrifugation. The cell pellets were resuspended in Buffer A (50 mM Tris-HCl, pH 7.4) and disrupted by sonication. The insoluble fractions were removed by centrifugation and recombinant proteins in soluble fractions were successively purified by nickel-nitrilotriacetic acid agarose (GE healthcare, USA) and *m*-aminophenylboronic acid agarose (Sigma-Aldrich, USA) (Kim et al., 2017). The eluted fractions containing the proteins were finally loaded on to a Superdex 75 HR 16/60 columns (GE healthcare, USA) equilibrated with Buffer A.

### Inhibition Assays

To evaluate inhibition activities of dinucleotides against class C β-lactamases, each enzyme (0.2 nM) was mixed with each dinucleotide (2 mM) in a 50 mM MES [2-(*N*-morpholino) ethanesulfonic acid] (pH 6.5) buffer. Then, nitrocefin (100 μM) was added to each solution and the nitrocefin hydrolysis was monitored at 486 nm for 1 h (SpectraMAX Plus, Molecular Devices, USA). The enzyme activity was quantified based on a percentage equation of [*v*_i_/*v*_0_ × 100], where *v*_i_ and *v*_0_ are the initial velocity of nitrocefin hydrolysis in the presence and absence of dinucleotides, respectively.

Four sets of rate experiments were carried out to determine the inhibition mechanism and the *K*_*i*_ value of NADPH. The double-reciprocal plots were generated by plotting the inverse initial velocity as a function of the inverse of the nitrocefin concentrations (10, 30, 40, 70, and 90 μM) in the presence of various concentrations of NADPH (0, 20, 100, and 250 μM). The plots were fitted to the competitive-inhibition equation.

(1) $$\begin{array}{r} v_{0}=\frac{v_{\max}\left[S\right]}{K_{m}\left(1+\frac{\left[I\right]}{K_{i}}\right)+\left[S\right]} \end{array}$$

where *v*_max_ is the maximum velocity of nitrocefin hydrolysis, [S] is concentration of nitrocefin, and [I] is the concentration of NADPH (Copeland, 2005). The reported value was calculated using the Origin software (OriginLab, USA).

### Protein-Ligand Binding Affinity Determination

The auto isothermal titration calorimeter (Auto-ITC) assay was performed using MicroCal Auto-iTC200 at Korea Basic Science Institute. AmpC BER (167 μM) and NADPH (2 mM) in the same buffer consisting of 20 mM HEPES pH 7.4 and 100 mM NaCl were loaded into the cell and the syringe, respectively. To detect thermo-changes during the enzyme-ligand interaction, 2 μL of NADPH was injected to the cell containing AmpC BER 19 times with 150 s intervals at 25°C. The collected titration curve was fitted to the one-set-of-sites interaction model of Origin software (OriginLab, USA).

### Reactivation Assays

The reactivation of inactivated AmpC BER was examined by a jump dilution method (Copeland et al., 2011). AmpC BER (4 μM) was completely inactivated by 10 mM NADPH or 500 μM adenosine 5′-(*P*-acetyl) monophosphate (acAMP) for 2 h at room temperature. To remove excess inhibitors, the inactivated AmpC BER proteins were diluted 8,000-fold in a 50 mM MES buffer (pH 6.5) and 100 μM nitrocefin without NADPH and acAMP. The reactivation of the diluted enzyme (0.5 nM) was monitored by measuring nitrocefin hydrolysis at 486 nm.

### Molecular Modeling of the AmpC BER/NADPH Complex

To dock NADPH into the active site of AmpC BER, water, sulfate, and AMP molecules were removed from the structure of AmpC BER (PDB code 5F1G). Then, polar hydrogen atoms were added to AmpC BER. The coordinates of NADPH were generated with LibCheck (Vagin et al., 2004) which is a ligand builder in Coot. Docking of NADPH was performed with AutoDock Vina (Trott and Olson, 2010) in the active site of AmpC BER.

### *In vitro* Antimicrobial Susceptibility Study

The *E. coli* clinical isolate BER (*E. coli* BER) producing AmpC BER was kindly provided by Dr. Patrice Nordmann. Ceftazidime **(**Sigma-Aldrich, St. Louis, MO) and NADPH **(**Sigma-Aldrich, St. Louis, MO) were dissolved in fresh distilled water for each experiment. *In vitro* antimicrobial susceptibility test was performed by using the microbial growth curve. For growth curve generation, 5 × 10^4^ colony-forming units (CFU) of the *E. coli* BER isolate were inoculated to 100 mL of Luria-Bertani (LB) broth containing ceftazidime and/or NADPH by indicated concentration and cultured in a rotary shaker at 37°C. The growth based on optical density at 600 nm was measured with 2 h intervals for 20 h of the assay.

To evaluate the minimum inhibitory concentration (MIC) of ceftazidime against the *E. coli* BER strain in combination with NADPH, we used a modified broth microdilution method. Briefly, bacterial suspensions were diluted to a density of 1 × 10^3^ CFU/mL in LB broth. The diluted bacteria (100 μL) were then added to 96 well-microtiter plates containing 100 μL of ceftazidime or ceftazidime with NADPH (417 μg/mL) and incubated for 14–15 h at 35°C. MICs were determined as the lowest concentration of the antibiotic agent at which there was no visible bacterial growth. These MIC titrations were performed at three independent experiments in duplicate.

### Ethics Statement for Animal Use

All mouse experiments were approved and performed according to the guidelines by the Institutional Animal Care and Use Committee at Chonbuk National University (Approved No. CBU 2014-0020 and CBU 2015-0040). All experiments were designed to reduce or minimize the numbers of animals used, and every effort was made to cause the minimum pain and distress to the animals.

### Animal Study

All wild-type female CD1 mice were purchased from a commercial source (Orient Bio Inc., a branch of Charles River Laboratory, Seongnam, Korea) and housed in specific pathogen-free unit. Age-matched female CD1 mice (4–5 weeks old) were intraperitoneally infected with 1 × 10^8^ CFU of *E. coli* BER strain producing AmpC BER and then treated with a single dose of ceftazidime (460 μg/mouse), and/or NADPH (15 mg/mouse) by intraperitoneal injection at 1 h after infection. The infected mice were followed by observation for more than 96 h.

### Statistical Analysis

We used Prism software (GraphPad Software, Inc., CA. USA) to analyze all data. Briefly, statistical analyses of inhibitory effects of nucleotides on class C β-lactamases were performed using a two-tailed paired Student's *t*-test, and growth curves were compared using an ANOVA with a multiple comparisons test. Kaplan-Meier survival curves were analyzed by the long rank test. A *P* < 0.05 was considered statistically significant differences.

## Results and Discussion

### NADPH Is a Competitive Inhibitor of AmpC BER

Recently, our studies demonstrated that GMP and IMP are non-covalent competitive inhibitors (Na et al., 2017) of class C β-lactamases and acAMP exerted its inhibitory effect through the covalent attachment of its AMP moiety to the nucleophilic serine residue of class C β-lactamases (Kim et al., 2017). Interestingly, the AMP moiety and GMP/IMP bind to R1 and R2 subsites in the active site of class C β-lactamases, respectively, although they have identical structures except for subtle differences in the purine base; the R1 and R2 subsites structurally refer to the distinct active site regions that accommodate the R1 side chain at the position C7 (or C6) and the R2 side chain at the position C3 (or C2) of β-lactam antibiotics on substrate binding, respectively (Kim et al., 2006). This observation suggested that the active site of class C β-lactamases composed of R1 and R2 subsites is probably large enough to accommodate two nucleotides.

**Figure 1:**
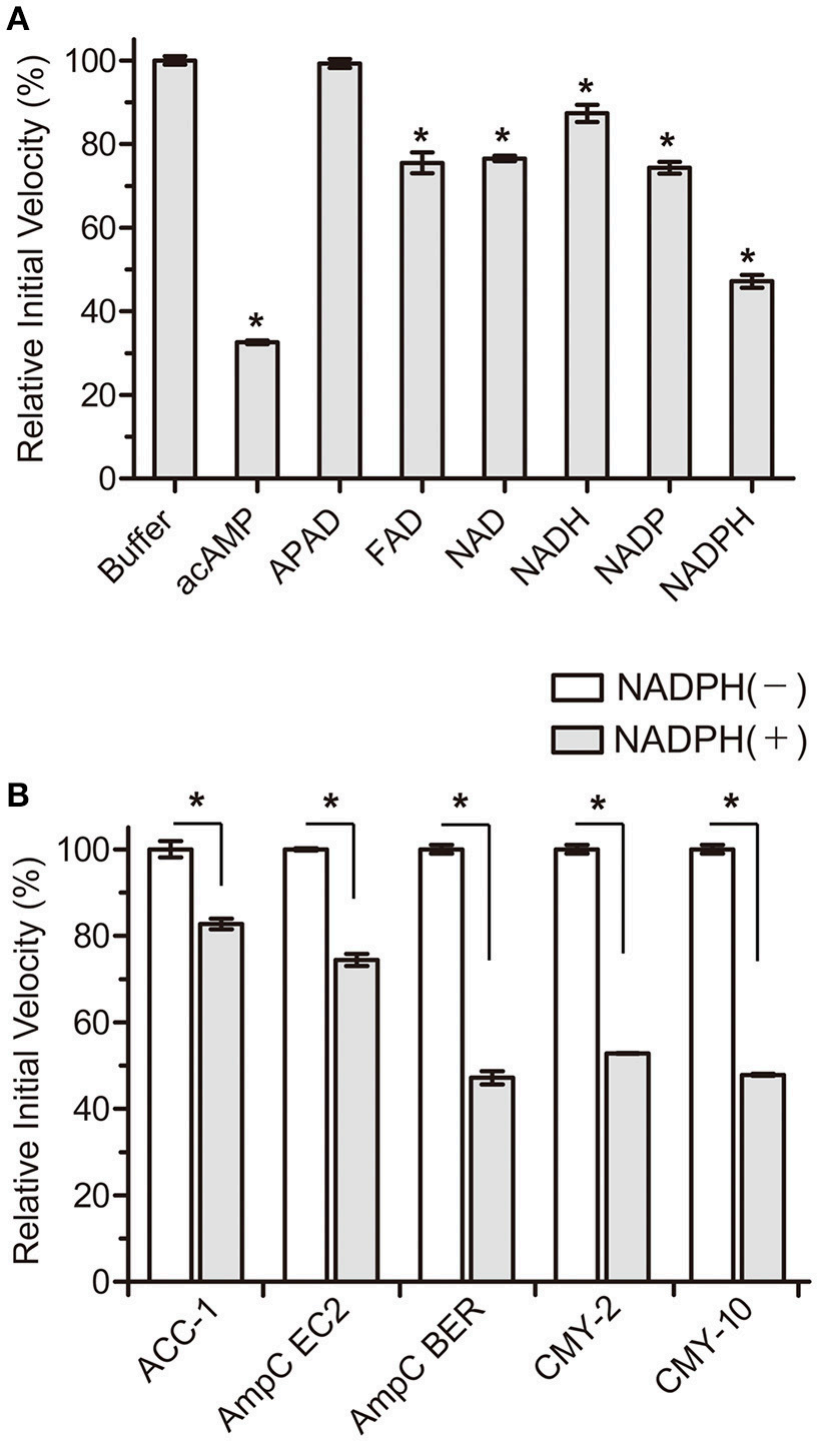
Effects of NADPH on the initial velocity of nitrocefin hydrolysis catalyzed by class C β-lactamases. All data represent the average of the three independent experiments. Error bars indicate standard deviations. **(A)** Percentage changes in the initial velocity of nitrocefin hydrolysis catalyzed by AmpC BER in the presence of nucleotides (2 mM). Abbreviation of nucleotides are as follows: adenosine 5′-(*P*-acetyl) monophosphate (acAMP), 3-acetylpyridine adenine dinucleotide (APAD), flavin adenine dinucleotide (FAD), β-nicotinamide adenine dinucleotide (NAD), β-nicotinamide adenine dinucleotide, reduced (NADH), β-nicotinamide adenine dinucleotide 2′-phosphate (NADP), and β-nicotinamide adenine dinucleotide 2′-phosphate, reduced (NADPH). **(B)** Percentage changes in the initial velocity of nitrocefin hydrolysis catalyzed by ACC-1, AmpC EC2, AmpC BER, CMY-2, and CMY-10 in the presence of NADPH (2 mM). Asterisks denote statistically significant differences with *P* < 0.05.

Based on these perspectives and β-lactamase structures, our strategy focused on adenosine dinucleotides and investigated the inhibitory efficiency of these dinucleotides against the AmpC BER β-lactamase from an *E. coli* clinical isolate (*E. coli* BER); AmpC BER is an extended-spectrum class C enzyme that can hydrolyze ceftazidime and other oxyimino cephalosporins (cefotaxime and cefepime) (Kim et al., 2006). Except for 3-acetylpyridine adenine dinucleotide (APAD), all tested adenosine dinucleotides including FAD, NAD, NADP, and NADPH reduced the initial velocity of nitrocefin hydrolysis catalyzed by AmpC BER (~15–55% inhibition, *P* ≤ 0.0184) when compared to a negative buffer control (Figure 1A). Notably, NADPH induced ~55% reduction in the initial velocity of nitrocefin hydrolysis, which is comparable to the initial velocity of nitrocefin hydrolysis in the presence of acAMP as a positive control. NADPH also reduced the initial velocity of nitrocefin hydrolysis catalyzed by other class C β-lactamases including ACC-1, AmpC EC2, CMY-2, and CMY-10 (Figure 1B). These results demonstrate that NADPH could confer inhibitory effect on various class C β-lactamases. In line with this result, the compatible activity of NADP dinucleotide has also been observed for another β-lactamase. Ogawara and Horikawa showed that NADP decreased the benzylpenicillin-hydrolyzing activity of β-lactamase from *Streptomyces cellulosae* although they did not evaluate the inhibition activity of NADPH (Ogawara and Horikawa, 1979).

**Figure 2:**
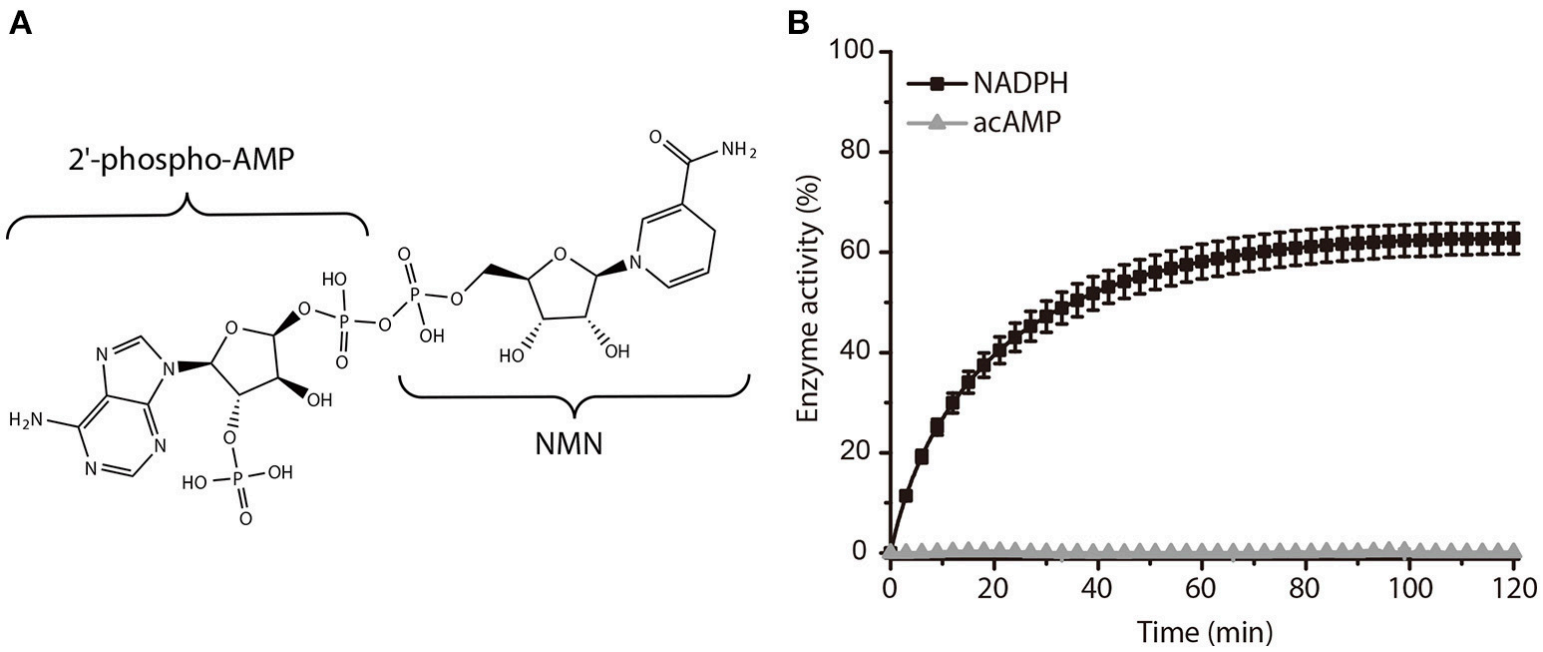
Activity recovery of AmpC BER inactivated by NADPH. **(A)** Chemical structure of NADPH. **(B)** Time course of AmpC BER reactivation. Error bars indicate standard deviations for triplicate experiments.

**Figure 3:**
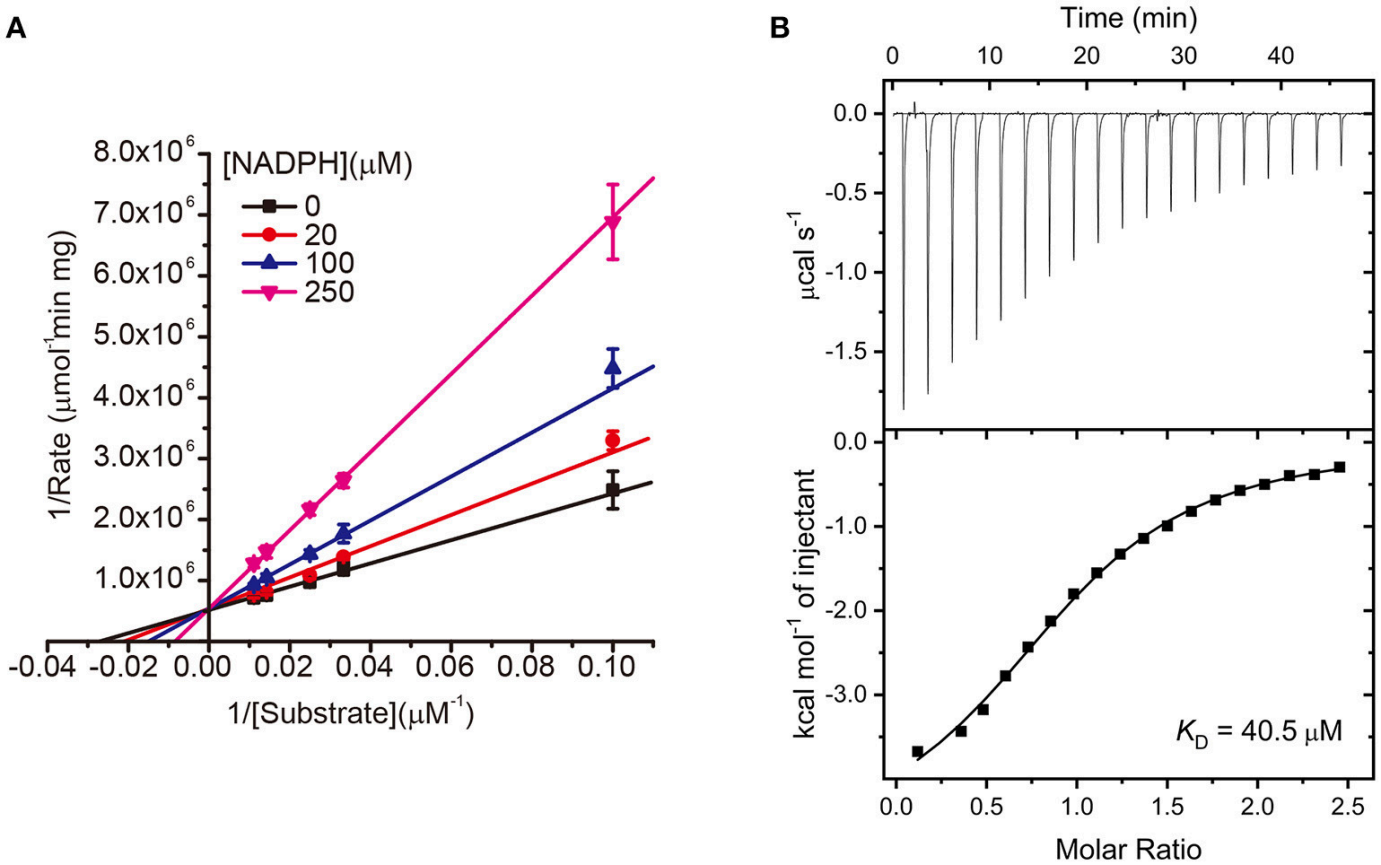
**(A)** Lineweaver–Burk plots of AmpC BER inhibited by NADPH. All data represent the average of the three independent experiments. Error bars indicate standard deviations. **(B)** ITC experiment to measure the binding affinity of NADPH toward AmpC BER. The graph placed on the top is the raw titration curve and the bottom one is the fitted data utilized to calculate the *K*_D_ value.

In a further study, we subsequently investigated the inhibition mode of NADPH against AmpC BER. According to a previous study (Kim et al., 2017), the AMP moiety of acAMP was covalently attached to the nucleophilic serine of AmpC BER in an irreversible manner. Since the 2′-phospho-AMP and nicotinamide mononucleotide (NMN) in NADPH were connected through the phosphoanhydride bond (Figure 2A), we speculated that the nucleophilic serine would attack one of the phosphorous atoms of the phosphoanhydride and the 2′-phospho-AMP or NMN moiety of NADPH might be covalently linked to the nucleophilic serine of AmpC BER with the NMN or 2′-phospho-AMP moiety as a leaving group, respectively. To test this possibility, we examine whether NADPH is an irreversible covalent inhibitor. Interestingly, AmpC BER inactivated by NADPH was reactivated in a time-dependent manner after the jump dilution (Figure 2B) whereas the reactivation of AmpC BER inactivated by acAMP was not detected as previously observed (Kim et al., 2017). Although more studies are required, it appears that the nucleophilic serine of AmpC BER couldn't attack the phosphorous atom of NADPH. As expected, the nucleophilic serine was revealed to be intact when AmpC BER inactivated by excess NADPH was analyzed by mass spectrometry (data not shown). Consequently, it is reasonable to assume that NADPH could inhibit class C β-lactamases in a non-covalent manner. We also performed steady-state kinetic analyses (Na et al., 2017) to determine the detailed inhibition mechanism of NADPH against AmpC BER. For these analyses, the double-reciprocal plots were generated by plotting the inverse initial velocity as a function of the inverse of the nitrocefin concentration in the presence of various concentrations of NADPH. Interestingly, *v*_max_ remained constant but the apparent value of *K*_m_ increased along with increasing NADPH concentration (Figure 3A), which indicates that NADPH acted as a competitive inhibitor of AmpC BER. The *K*_*i*_ value of NADPH toward AmpC BER was determined to be 103 μM. Furthermore, the dissociation constant (*K*_D_) of NADPH toward AmpC BER measured by isothermal titration calorimeter (Figure 3B) was 40 μM. Taken together, our data suggests that NADPH is a non-covalent competitive inhibitor of class C β-lactamases.

### Molecular Docking Simulation of the AmpC BER/NADPH Complex

**Figure 4:**
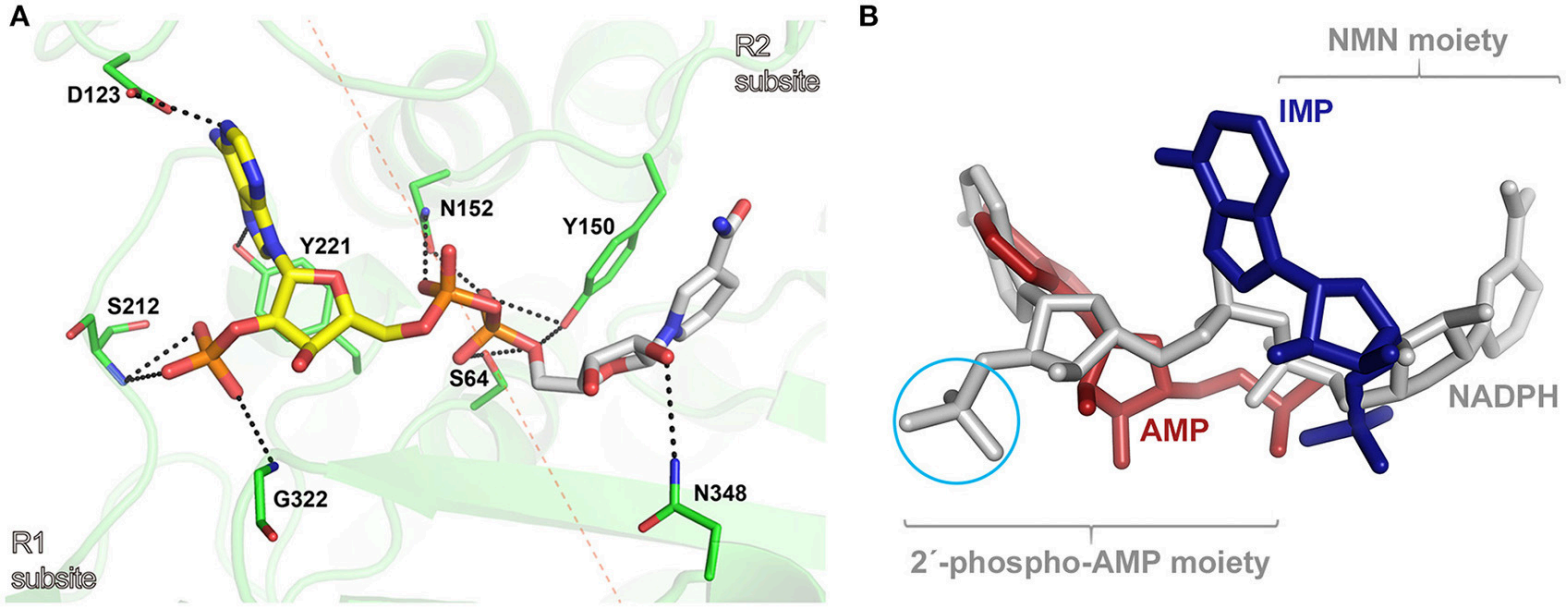
**(A)** Molecular docking model of the AmpC BER/NADPH complex. The active site of AmpC BER is shown as a transparent cartoon. A dotted red line crossing the nucleophilic seine (Ser64) indicates the border between R1 and R2 subsites. The active site residues (green) and NADPH (yellow: 2′-phospho-AMP moiety, gray: NMN) are represented by sticks. Residues interacting with NADPH are numbered according to the canonical numbering of *Enterobacter cloacae* P99 AmpC (Galleni et al., 1988). Dotted black lines indicate electrostatic interactions. **(B)** The structural superposition among the AmpC BER/NADPH complex, the CMY-10/IMP complex, and the adenylylated AmpC BER. NADPH, IMP, and AMP are colored in gray, blue, and red, respectively. A cyan circle indicates the 2′-phosphate group in the ribose of NADPH. For clarity, lactamases are not presented.

To get insights into the binding mode of NADPH in the active site of AmpC BER, we tried to solve the crystal structure of the AmpC BER/NADPH complex. However, our efforts to grow crystals of the complex has not been successful so far. In parallel with crystallization experiments, the *in silico* complex model was generated by docking NADPH into the active site of AmpC BER by using the AutoDock Vina program (Trott and Olson, 2010). According to the complex model, the 2′-phospho-AMP moiety exclusively interacts with residues in R1 subsite (Figure 4A). The pyrimidine ring and the imidazole ring in the adenine base make hydrogen bonds with Asp123 and Tyr221, respectively. The ribose is packed against Tyr221 with its 2′-phosphate group interacting with the backbone -NH groups of Ser212 and Gly322. The 5′-phosphate group of the ribose ring interacts with Asn152. However, the NMN moiety forms fewer interactions with residues in R2 subsite (Figure 4A). The nicotinamide ring stacks against Tyr150, the 2′-OH of the ribose is hydrogen bonded to Asn348, and the 5′-phosphate group interacts with the nucleophilic Ser64, Tyr150, and Asn152.

The *in silico* AmpC BER/NADPH complex model provides structural explanation why NADPH displayed a stronger inhibition activity than NADH and its oxidized form, NADP. The lower inhibition activity of NADH can be attributed to the absence of 2′-phosphate in the ribose considering multiple interactions between the phosphate group in NADPH and active site residues (Figure 4A). The structural differences between NADP and NADPH are the aromaticity and the number of hydrogen atoms in the nicotinamide ring. Some proteins can discriminate between NADP and NADPH. For example, in *Aspergillus nidulans* NmrA, a transcription repressor involved in the regulation of nitrogen metabolism, those subtle differences in the nicotinamide ring were suggested to affect ring stacking interactions with the side chain of a tyrosine residue, contributing to the discrimination between NADP and NADPH (Lamb et al., 2003). It is notable that the nicotinamide ring of NADPH forms stacking interactions with the side chain of Tyr150 in our AmpC BER/NADPH complex model. The different inhibition activity of NADP and NADPH might be related to their different stacking interactions with Tyr150.

The *in silico* AmpC BER/NADPH complex model was superposed onto crystal structures of the adenylylated AmpC BER and the CMY-10/IMP complex. As shown in Figure 4B, the binding mode of NADPH is different from those of the adenylate covalently linked to the nucleophilic serine of AmpC BER and IMP. The 2′-phospho-AMP moiety of NADPH has an additional phosphate group compared to the adenylate and the single ring structure of the NMN moiety of NADPH is different from the double ring structure of IMP. The covalent linkage between the two moieties, together with structural disparity, is likely to affect interactions of NADPH with active site residues.

### *In vitro* and *in vivo* Activity of NADPH in Combination With Ceftazidime Against the *E. coli* BER Strain

**Figure 5:**
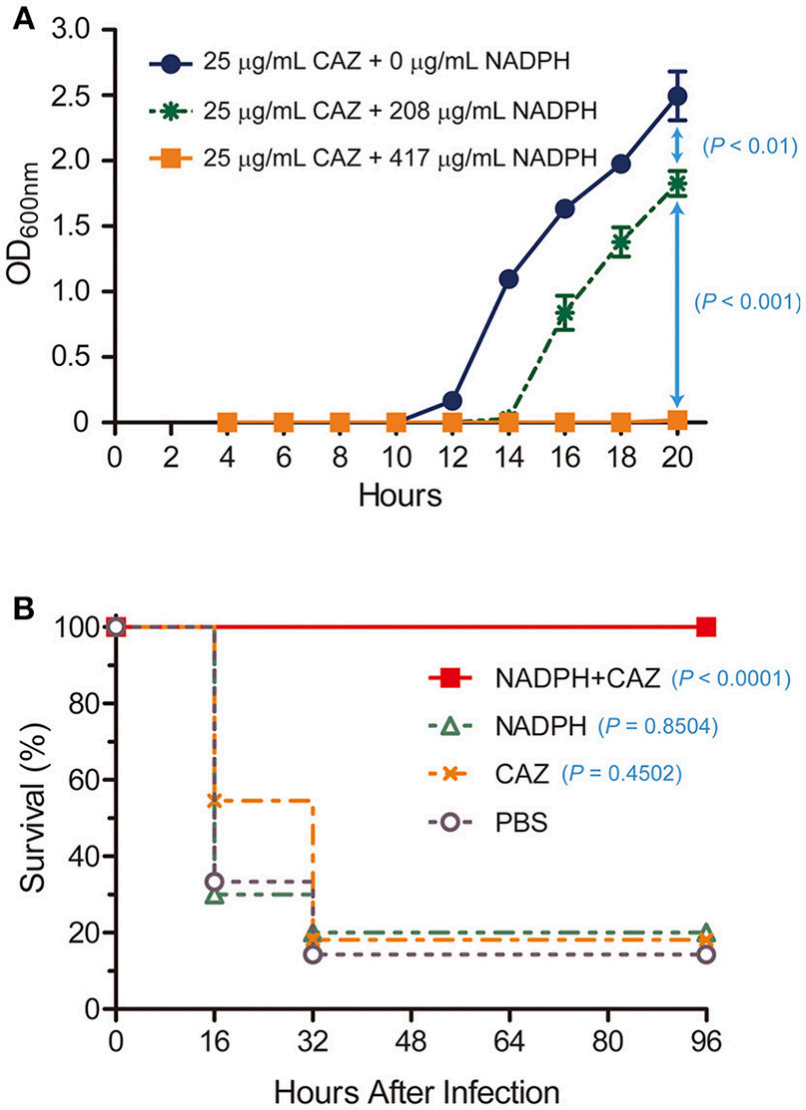
*In vitro* and *in vivo* restoration of susceptibility of ceftazidime-resistant bacteria by NADPH. **(A)** *In vitro* combined effect of NADPH and ceftazidime (CAZ) on a CAZ-resistant *E. coli* BER strain producing AmpC BER. The growth curves were constructed using data from three independent experiments. Error bars indicate standard deviations. **(B)** Rescue of ceftazidime (CAZ) activity by NADPH in a mouse model. Survival rates were represented as percentage from at least two independent experiments, each with five to six mice.

Based on our observation that NADPH was a competitive inhibitor of AmpC BER and nicely fit into the active site of AmpC BER in the modeled structure, we postulated that NADPH could be exploited to restore the antibiotic activity of ceftazidime toward the *E. coli* BER strain by inhibiting AmpC BER. To perform the *in vitro* antimicrobial susceptibility test based on growth rate, we determined whether ceftazidime in combination with NADPH could inhibit the growth of the ceftazidime-resistant *E. coli* BER strain (Figure 5A). Interestingly, the *E. coli* BER strain in the media containing both NADPH (208 μg/mL and 417 μg/mL) and ceftazidime (25 μg/mL) displayed significantly lower growth rates than the bacteria in the media only containing ceftazidime (25 μg/mL) (*P* < 0.01). The growth inhibitory effect of NADPH in combination with ceftazidime was dose dependent and the combination of 417 μg/mL NADPH and 25 μg/mL ceftazidime completely inhibited the growth (*P* < 0.001) until 20 h after seeding with 5 × 10^4^ CFU of the *E. coli* BER strain. However, NAPDH alone did not show any effect on the growth rate of the *E. coli* BER strain (data not shown). Supportively, the MIC of ceftazidime against the *E. coli* BER strain was reduced from 32 μg/mL in the absence of NADPH to 8 μg/mL in the presence of 417 μg/mL NADPH.

As the ceftazidime/NADPH combination effectively reduced the growth of the *E. coli* BER strain *in vitro*, we further investigated whether NADPH would provide therapeutic benefit *in vivo* against the ceftazidime-resistant bacteria through reversing AmpC BER-mediated resistance to this antibiotic. To evaluate this potential, post-exposure treatment experiments were performed in a mouse infection model (Figure 5B). Age-matched female CD1 mice, 4–5 weeks old, were intraperitoneally inoculated with a lethal dose (1 × 10^8^ CFU) of the *E. coli* BER strain. At 1 h after infection, a single dose of ceftazidime (460 μg/mouse), NADPH (15 mg/mouse), or ceftazidime (460 μg/mouse) in combination with NADPH (15 mg/mouse) was treated into the infected mice through intraperitoneal injection. Notably, ceftazidime in combination with NADPH demonstrated the marked protective efficacy against the ceftazidime-resistant bacteria (100% survival rate, *P* < 0.0001) compared to the saline (PBS)–treated control (~14% survival). In contrast, a single dose of either ceftazidime or NADPH did not provide significant benefit on survival (ceftazidime, ~18% survival rate, *P* = 0.4502; NADPH, 20% survival rate, *P* = 0.8504). Furthermore, when non-infected CD1 mice were intraperitoneally administrated with ceftazidime alone or NADPH alone, no detectable effects were observed on survival rate (100% survival rate; data not shown). Taken together, these findings suggest that the combinatorial treatment of ceftazidime with NADPH could resensitize the ceftazidime-resistant *E. coli* BER strain to ceftazidime through inhibiting the activity of AmpC BER β-lactamase, thereby preventing mice from lethal infection caused by the AmpC BER-producing *E. coli* strain. However, we acknowledge that the effective dose of NADPH observed in our primary study might be high but could be reduced through the chemical optimization to enhance the binding affinity toward β-lactamases. In summary, although NADPH is metabolic dinucleotides that are widely used as cofactors or substrates in living organisms for redox reactions, anabolic pathways, mitochondrial functions, calcium homeostasis, and aging (Sohal et al., 1990; Krause, 2007; Spaans et al., 2015), we demonstrated that NADPH could be a competitive inhibitor of a class C β-lactamase, AmpC BER, and the combinatorial treatment with ceftazidime and NADPH provided significant antibacterial efficacy against the ceftazidime-resistant *E. coli* BER strain producing AmpC BER. Taken together, our findings suggest that dinucleotide scaffolds could lead to novel β-lactamase inhibitors that may have clinical potential as non-toxic inhibitors.

## Author Contributions

KC and S-SC designed experiments, analyzed data, and wrote the manuscript. J-HN, TL, S-BP, M-KK, and B-GJ performed experiments.

### Conflict of Interest Statement

S-SC, J-HN, KC, TL are named inventors on patents for the use of NADPH and its derivatives as β-lactamase inhibitors. The remaining authors declare that the research was conducted in the absence of any commercial or financial relationships that could be construed as a potential conflict of interest.
